# Infant respiratory syncytial virus infection and childhood asthma: A shift in the paradigm?

**DOI:** 10.1002/ctm2.1414

**Published:** 2023-09-12

**Authors:** Christian Rosas‐Salazar, Tina V. Hartert

**Affiliations:** ^1^ Department of Pediatrics Vanderbilt University Medical Center Nashville Tennessee USA; ^2^ Department of Medicine Vanderbilt University Medical Center Nashville Tennessee USA

The idea that some non‐respiratory viruses can impact health years after the initial infection is well established.^1^ For example, someone acutely infected with hepatitis B or human papillomavirus can develop a chronic infection that may result in cirrhosis or cervical cancer, respectively, decades later. Whether RNA respiratory viruses (e.g. respiratory syncytial virus [RSV], influenza virus, parainfluenza virus, metapneumovirus, rhinovirus and coronavirus) result in chronic morbidity has been more difficult to study and establish. This is in part because —unlike hepatitis B or human papilloma virus— most RNA respiratory viruses don't become chronic infections, generally cause a mild disease, and don't lead to persistent symptoms in most infected individuals. Thus, despite the recognized global burden associated with RNA respiratory viruses, acute respiratory infections due to these infectious agents have been traditionally thought of as a transient illness with no important long‐term effects.^2^, ^3^ However, emerging evidence of potentially causal associations between RNA respiratory viruses and the onset of chronic lung diseases (e.g. severe acute respiratory syndrome coronavirus 2 and post coronavirus disease [post‐COVID] conditions or human rhinovirus C and childhood asthma) has shifted this paradigm, suggesting that these infectious agents are not merely “in‐and‐out” and may cause more than an acute self‐limited disease.^4^, ^5^ This is of significant importance in understanding host risk factors, identifying viral strains resulting in chronic disease, and in considering prevention of long‐term complications.

## 1 DOES INFANT RSV INFECTION CAUSE CHILDHOOD ASTHMA?

Respiratory syncytial virus is the most common etiology of lower respiratory tract infections (LRTIs) in infants worldwide, although it usually causes asymptomatic or mild disease in the majority of infants.^3^ The potential association between infant RSV LRTIs and childhood asthma was first described in 1959.^6^ Since then, numerous observational studies and meta‐analyses have repeatedly demonstrated that children with an early‐life RSV LRTI are at a higher risk of childhood asthma.^7^, ^8^, ^9^, ^10^ However, an RSV LRTI is not an exposure, it is a clinical phenotype. In addition, because RSV LRTIs and childhood asthma share many clinical features (such as wheezing) and could have a shared genetic risk, an RSV LRTI in infancy may just represent the first acute asthma expression in a genetically predisposed child.^7^, ^11^, ^12^ To address this issue, we designed a prospective birth cohort of ∼2000 unselected, healthy term children to test the association of infant RSV infection (and not solely infant RSV LRTI) with childhood asthma.^13^ This study design has the advantage of avoiding confounding by a shared genetic origin, as we have found no evidence of genetic risk for infant RSV infection.^14^ Furthermore, this study design gives us the opportunity to study the group of children not infected with RSV in infancy to understand the impact of delaying the infant RSV infection on childhood asthma risk. In contrast, studies focusing on children with an infant RSV LRTI compare this group to those without an infant RSV LRTI, although the latter is a heterogenous group that includes children not infected with RSV in infancy and those who had milder RSV infections in the first year of life. In our study, using molecular and serologic testing to establish RSV infection or no infection during infancy, we found that ∼54% of children are infected with RSV in infancy, which is consistent with other studies.^15^, ^16^ Importantly, children without an infant RSV infection had a ∼26% lower risk of childhood asthma than those with an infant RSV infection. In exploratory analyses, infant RSV infection was more strongly associated with development of a non‐atopic asthma phenotype. Last, leveraging the population‐based study design that included children infected and not infected with RSV in infancy, we estimated that ∼15% cases of childhood asthma could be prevented by delaying RSV infection until after the first year of life. Taken together, our findings suggest a causal effect of infant RSV infection on childhood asthma, although we recognize that causality cannot be definitively established in observational studies. However, there is accumulating evidence from multiple observational studies, animal and in vitro models, and one randomized controlled trial of RSV monoclonal antibodies that provide multi‐level support for a causal association between infant RSV infection and childhood asthma.^7^, ^8^, ^9^, ^10^, ^17^, ^18^

## 2 HOW CAN INFANT RSV INFECTION CAUSE CHILDHOOD ASTHMA?

Intuitively it might seem that a severe insult to the developing lungs and immune system (such as an infant RSV LRTI) would be involved in the onset of childhood asthma, but the vast majority of children in our study actually had a mild or asymptomatic RSV infection.^13^ Studies from our group indicate that even mild infant RSV infections are associated with anti‐viral immune dysregulation and abnormal airway cell metabolism that can persist for several years.^19^, ^20^ This differential early impact of infant RSV infections is supported by murine models demonstrating impaired regulatory T cell function among mice with early‐life RSV infections.^17^ In chronic diseases associated with non‐respiratory viruses, certain viral strains are associated with differences in both the severity of the acute infection and the risk of developing long‐term complications. Limited data suggest that certain RSV strains can result in more severe disease, can lead to differential host immune response, and may be more likely to result in viral persistence.^14^, ^21^, ^22^, ^23^ We speculate these factors could result in chronic airway inflammation and/or reprogramming of the developing airway epithelium as pathways through which infant RSV infection contributes to childhood asthma onset. Lastly, we have shown that infant RSV infection is associated with an airway microbiome pattern that has been associated with childhood asthma risk.^24^

## 3 WHAT ARE THE IMPLICATIONS OF OUR FINDINGS?

There are currently no established methods for the primary prevention of childhood asthma, although our results suggest that this could be achieved through the delay of infant RSV infection. The implications of this are enormous, as infant RSV infection and childhood asthma are among the most common acute and chronic lung diseases in the pediatric population, respectively. The profound near elimination of RSV diseases during the initial phase of the COVID‐19 pandemic demonstrates that the prevention of infant RSV infection is possible through public health measures, which include non‐pharmaceutical interventions.^25^ Recently, randomized controlled trials of long‐acting RSV monoclonal antibodies and maternal RSV vaccines have been shown to decrease the severity of infant RSV infections (thus preventing infant RSV LRTIs), although it is unknown if these products also prevent infection with this RNA respiratory virus.^26^, ^27^ Our findings highlight the importance of observational studies taking advantage of post‐approval vaccine uptake and following up with participants enrolled in randomized clinical trials of long‐acting monoclonal antibodies and maternal vaccines until older ages to ascertain childhood asthma‐related outcomes, recognizing that these trials cannot remain blinded. Our study results also add to emerging literature demonstrating that RNA respiratory viruses may lead to substantial long‐term adverse respiratory and other health effects, phenomena that we need to understand to be able to prevent and treat chronic diseases that follow these common infections.

## CONFLICT OF INTEREST STATEMENT

Christian Rosas‐Salazar serves as a consultant for Amgen and AstraZeneca. Tina V. Hartert has served on vaccine Data Safety Monitoring Boards for Pfizer and as a consultant for Sanofi‐Pasteur.
